# Histopathology: ditch the slides, because digital and 3D are on show

**DOI:** 10.1007/s00345-018-2202-1

**Published:** 2018-02-02

**Authors:** Ilaria Jansen, Marit Lucas, C. Dilara Savci-Heijink, Sybren L. Meijer, Henk A. Marquering, Daniel M. de Bruin, Patricia J. Zondervan

**Affiliations:** 1 Department of Urology, Academic Medical Center, Meibergdreef 9, 1105 AZ Amsterdam, The Netherlands; 2 Department of Biomedical Engineering and Physics, Academic Medical Center, Amsterdam, The Netherlands; 3 Department of Pathology, Academic Medical Center, Amsterdam, The Netherlands; 4 Department of Radiology, Academic Medical Center, Amsterdam, The Netherlands

**Keywords:** Digital pathology, Three-dimensional, Computer-aided diagnosis, Urinary tract pathology

## Abstract

Due to the growing field of digital pathology, more and more digital histology slides are becoming available. This improves the accessibility, allows teleconsultations from specialized pathologists, improves education, and might give urologist the possibility to review the slides in patient management systems. Moreover, by stacking multiple two-dimensional (2D) digital slides, three-dimensional volumes can be created, allowing improved insight in the growth pattern of a tumor. With the addition of computer-aided diagnosis systems, pathologist can be guided to regions of interest, potentially reducing the workload and interobserver variation. Digital (3D) pathology has the potential to improve dialog between the pathologist and urologist, and, therefore, results in a better treatment selection for urologic patients.

**Electronic supplementary material:**

The online version of this article (10.1007/s00345-018-2202-1) contains supplementary material, which is available to authorized users.

## Introduction

**Fig. 1:**
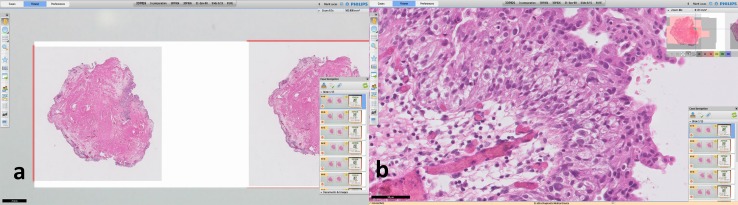
Overview of the Philips information management system. **a** Showing an overview of a case, an en-bloc resection of a bladder tumor; **b** showing the zoomed in version, focusing on the papillary tissue

Regardless increase of cervical hernias and repetitive strain disorders, the conventional light microscope and the pathologist seem to remain inseparable partners [1]. Yet, recent developments in the field of digital pathology urge many pathology departments to digitize slides, enabling digital visualization (Fig. 1). Clearly, hospitals benefit from digital patient management systems and fully digitized radiology departments and even allow urologist to plan intrarenal surgeries with special 3D software [2]. Likewise, the digitization of pathology specimens will improve accessibility within the hospital and could facilitate multidisciplinary meetings, allowing oversees consultation from specialized pathologists.

Histopathologic analysis using the conventional light microscopy has been the gold standard for cancer detection and grading for decades. In general, of every tissue block arriving at the pathology department, only 1–2 slides per tissue block are assessed [3]. Therefore, it can be difficult for the pathologists to fully understand the growth pattern of a tumor [4]. It can be hypothesized that tumor invasiveness may vary from section to section. Moreover, most tumors are heterogeneous [5]. By assessing a small number of slides, an undersampling error is introduced. A method to sample a whole specimen holds the promise to provide the pathologists with a more accurate understanding of the growth pattern of a tumor [6].

Due to recent developments, two-dimensional (2D) digital microscopy images can be acquired through digital microscope systems. A method to visualize the whole specimen is to cut multiple consecutive 2D sections and create a three-dimensional (3D). Nonetheless, this approach has not been considered by pathologists to examine histology yet, mostly due to the effort and time involved in the preparation of the physical slides.

It is debatable whether the diagnostic accuracy would improve by increasing the number of digital slides. Histopathological examination is already laborious and susceptible for human variation [7]. By presenting even more data within the same time-span, more errors are prone to occur. To reduce the workload and inter-observer variation, computer-aided diagnosis (CAD) systems might be the solution. A CAD system could diminish the workload by automated recognition of suspicious tissue and guide the pathologist in the grading and staging of a tumor.

## The current workflow in pathology

The standard workup for a histology specimen is a labor-intensive and time-consuming process (supplementary Figure 1). At the end of this process, the pathologist examines the slides by looking at morphological changes. Using different magnifications, the aggressiveness of tumor cells is graded, and the staging is performed. Over the years, guidelines have been adjusted to improve prognostic information, helping the urologist in their treatment planning. However, histopathology remains notorious for its interobserver variability. In prostate cancer, interobserver studies show an agreement ranging from 10 to 70% when assessing the Gleason score [8]. In 20% of patients, this would have influenced the treatment plan [9]. While the bladder cancer grading system has a large prognostic value, the interobserver agreement is only 60% [10]. Diagnostic accuracy has been seen to improve when assessed by specialized urinary tract pathologist [11].

## 2D digital pathology

The Food and Drug Administration (FDA) only recently gave approval for the first digital histology slide scanner to be used for diagnostic purposes in the US [12], while it was already being used in various places in Europa and Canada. By optically scanning the histologic glass slide, a 2D ultra-high-resolution digital image is created, a so-called whole slide image (WSI). These WSIs can be visualized on a digital screen, making it possible to examine the image at different magnifications (see Fig. 1). Several studies proved the non-inferiority of WSIs for diagnostic purpose by comparing them with state-of-the-art light microscopy [13].

Even though digital pathology is available, it is not yet broadly implemented in the current clinical practice. Pathologists could be reluctant, since it requires another way of working. As of 2016, the College of American Pathology has issued a set of preliminary guidelines for digital pathology to anticipate the digital era [14]. To incorporate digital pathology, however, the workflow on the pathology department must be adjusted (supplementary Figure 1). It requires investments in WSI scanners, high-performance computers, high-quality color-calibrated monitors, and server solutions for data storage. WSIs consist of an enormous amount of data, depending on the size of the section; the storage size of a WSI can range from 1 to 5 GB for a non-compressed single prostate biopsy. This requires an enormous increase in computational power and network infrastructure.

If digital pathology is implemented correctly, it will lead to reduced costs, e.g., by balancing the workload or tele-consulting of (distant) specialists. For the implementation of digital pathology, it has been estimated that for a large academic institution with 219,000 annual accessions, a shift from conventional to WSIs would save of US$ 18 million over a 5-year period [15]. Table 1 gives a more detailed overview of benefits and barriers for the implementation of digital pathology.

**Table 1:** Benefits and barriers of implementing digital pathology

Benefits whole slide imaging	Barriers for adoption whole slide imaging
Accessibility and access by multiple observers	Change in ergonomics
Teleconsultations	Need for high-quality scanners
Eligible for CAD systems	Need for high-speed network
Possibility placing annotations and comments	Large size digital files
Sharing slides for research purposes	Costs: hardware, software, information technology support/infrastructure, and maintenance
Digital storage	Lack of standards and/or best practice guidelines
Portability and flexible work schedules	Scanning artifacts
Archiving interesting cases	
Enhancing workflow	
Integrated into pathology report/patient information system	
Pathology education	

## Three-dimensional (3D) reconstructions

Digital pathology could accelerate consultations and can replace the bright field microscope for education and clinical conferences [16]. A more recently studied application is the stacking of multiple 2D slides to reconstruct a 3D volume. This enables the pathologist to assess the resected tissue or biopsy as a whole. Orientation on single 2D slides is often difficult due to tangential sectioning and artifacts [17]. A 3D reconstruction can provide improved insight into the architectural features and spatial arrangements with other structures.

However, a major difficulty in the 3D representation is the alignment of the slides, since non-linear deformation occurs during the sampling process of fixating, sectioning, and mounting of the specimen [6, 18–21].

**Fig. 2:**
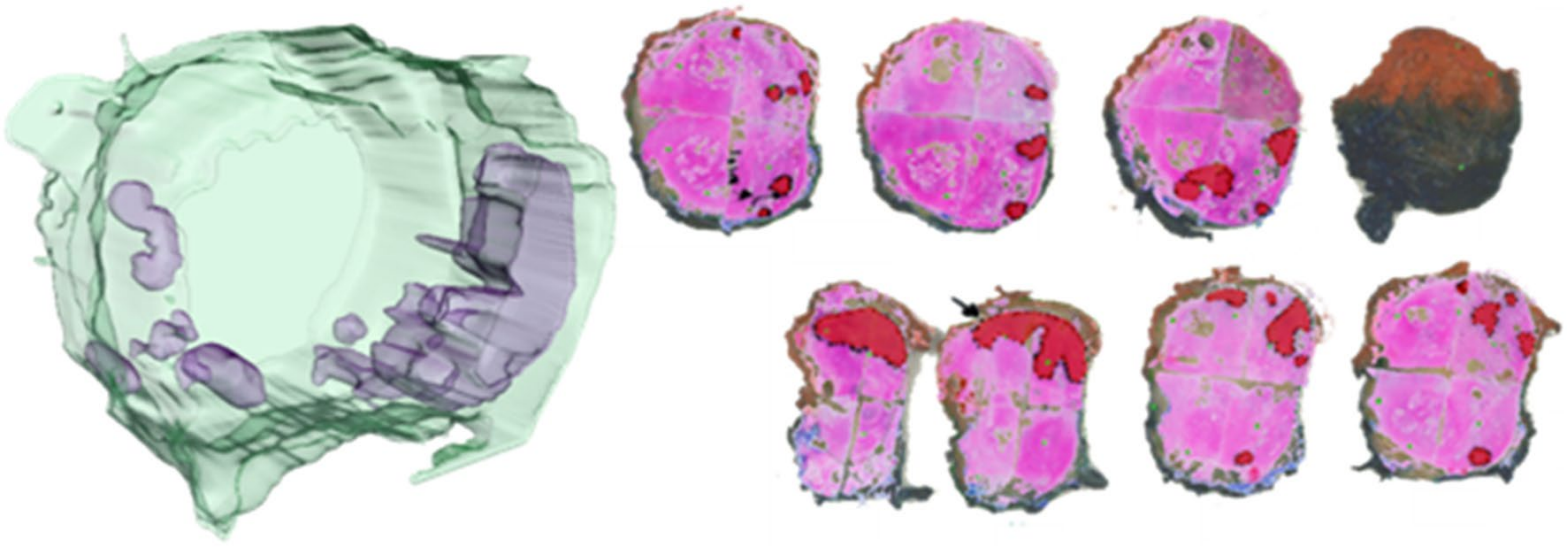
3D reconstruction of a prostatectomy specimen. On the right, the individual whole mount slides are shown in red the manually delineated tumor. From this, the 3D reconstruction on the left is rendered

Several studies have focused on the 3D reconstructed histology [6, 18–24]. Boag et al. reconstructed prostate carcinomas out of multiple 2D histological slides and segmented the adenocarcinoma to visualize the architecture of the tumorous glands. They found that Gleason grade 3 glands appear separate from each other on the 2D slides, while the 3D reconstruction showed interconnecting tubules [22]. Muller et al., impart of a correlation study, visualized Gleason grade 3 and 4 tumors in a 3D representation of a whole prostate, using 4 mm spaced WSIs (see Fig. 2) [25, 26].

For the visualization of 3D reconstructions, most studies used manual segmentations of tissue structures. As a next step, Norton et al. showed an automated method for the creation of 3D segmentations of in situ disease of breast tissue [21]. These automated segmentations could alleviate the laborious task of manual segmentations. 3D reconstructions are not solely based on H&E stained tissue. Some studies have shown that registration is also possible with other staining agents or even combined multiple stains or different modalities [6, 18, 19, 23].

The main drawback of these studies is the low out-of-plane resolution of 3D reconstructions. The size of these high-resolution data sets visualizing them in 3D is challenging, especially when looking at a higher magnification. The bias could be that you lose the details which could be of clinical relevance.

## Computer-aided diagnosis (CAD)

**Fig. 3:**
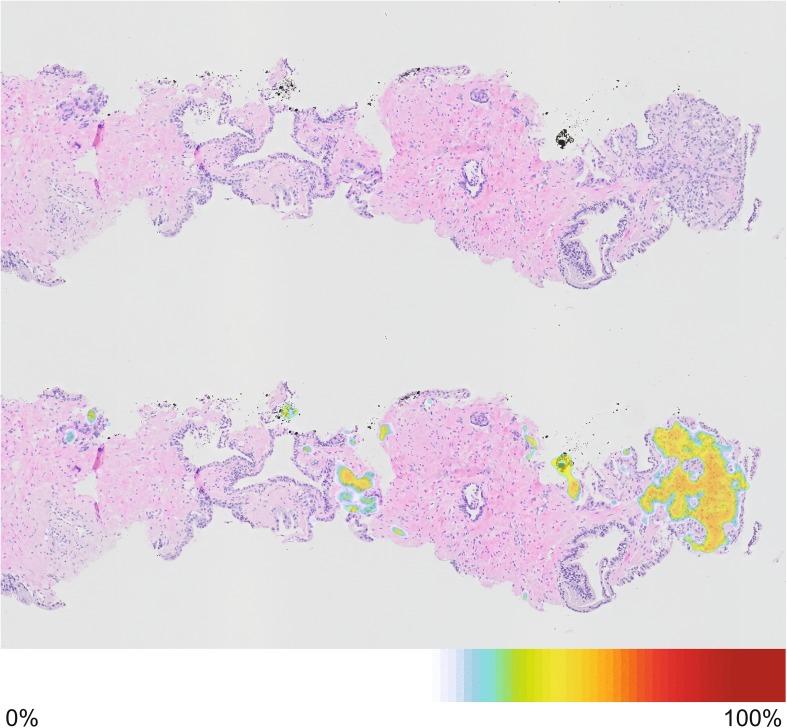
Digital prostate needle biopsy. Top image shows the normal H&E stained digital image with on the right and on the left (top) a focus of adenocarcinoma. Lower image shows the heatmap overlay indicating the probability of prostate cancer

Several solutions have been sought to handle the increase in workload and to reduce the existing observer variation of pathologists. Since the possibility to digitize histology slides, groups have applied automated pattern recognition software to identify suspicious features. The so-called CAD systems have been introduced to support pathologist in their decision-making [27]. These systems can automatically measure the extent of cancerous area, the grade of the cancer, and generate localized cancer maps (Fig. 3).

Initially, CAD systems used mathematical algorithms based on structural hand-crafted features or variations in pixel intensities [27]. Currently, the majority of CAD systems focus on prostate biopsies and high accuracy levels are found in the detection of prostate cancer [27]. In the grading, however, these systems seem less accurate. An explanation may be the focus on the glandular structures, where the adenocarcinoma originates. In high-grade tumors, there is an absence of glands and loss of differentiation [28] and thus no structures to detect.

More recently, deep learning has become more popular. Deep learning is often used for image recognition; for example, the automated classification of skin lesions in dermatology [29]. By applying a unique set of filters, it is possible to differentiate between preselected groups, for example different tumor grades. Litjens et al. have used deep learning in prostate biopsies and accomplished an accuracy of 93% in differentiating tumorous tissue from benign tissue [30]. Since most interobserver variation exists in the grading and staging of a tumor [7], it would be of great asset to train CAD systems for this application.

The major drawback of these deep learning techniques is the need of large annotated training sets. Preferably, these training sets should be assessed by multiple pathologists to reduce the influence of the interobserver variability. At this moment, there is a lack of multi-institutional validation sets to compare the performance of different CAD systems.

Ideally, a CAD system should be used to guide pathologists to a region of interest or even replace pathologists, certainly with the increased number of slides in 3D reconstructions. By doing so, communication, and thus interpretation, regarding the clinical information and the histopathological input could lead to better understanding of the tumorous tissue.

## Future

Currently, the use of histological 3D reconstructions in diagnostics is not feasible for clinical decision-making. Despite the major progress in digital pathology, there are still some hurdles to take. However, a set of challenges are already addressed to enable the future use of 2D and 3D digital pathology.

First, the sample workup for histopathology remains a labor-intensive process. To improve this, Onozato et al. have developed an automated tissue-sectioning machine, automating the sectioning and mounting of the histological sections [31]. At this moment, automated sectioning requires more time than the manual sample workup. However, it is the expectancy that eventually these systems can alleviate the manual work.

Although formalin is still the most common fixative, it may not always be the most optimal. The deleterious effects on DNA and RNA are well known, and proteins are altered by the crosslinking mechanism. An optimal fixative should allow for high-quality histology, preserve sufficient material for analysis using other technical approaches, and desirable, faster than the 1 mm/h penetration rate of formalin [32].

Clearly, 2D and 3D digital pathology is potentially a powerful tool for the pathologist. It has the opportunity to reduce workload, which has been increasing due to increasing number of requested diagnosis and a reduced number of pathologists [33]. It allows pathologists to set a diagnosis from anywhere in the world, and more important, it gives the possibility to consult distant colleagues. These teleconsultations can facilitate the second opinions without the risk-off slide loss. Teleconsultations can be of tremendous value for peripheral hospitals, giving the opportunity to easily consult urinary tract pathologists. Sub-specialization in urinary tract pathology is relatively rare and most pathologists work on a large variety of tissue types. Digital pathology has the potential to amplify sub-specialization and, therefore, limit the variation in diagnosis [34].

Although current CAD systems have high levels of accuracy for the detection of prostate cancer, there is no implementation into clinical practice yet. Most CAD systems are only validated in a single center, while, due to staining differences between the histological slides in different pathology laboratories, staining differences must be incorporated. Therefore, before these CAD systems can be used in daily practice, multi-institutional validation has to be performed by specialized pathologists.

By implementing 3D reconstructions together with CAD systems, pathologists will get a better understanding of the growth pattern of a tumor and can be more easily guided to specific regions of interest. By letting the pathologist focus on only the difficult cases, the workload diminishes.

Finally, by implementing digital pathology into the electronic patient information systems, they are readily available for urologist and, therefore, give easily access during multidisciplinary meetings or for intercollegial consultations. The possible 3D pathology image could be of clinical relevance for urologists. With the ongoing evolution in better imaging technologies, such as MRI and PSMA-PET scan, we hope that integration of these technologies together can help the urologist in optimizing treatment options. Subsequently, with better pathological and radiological mapping of prostate cancer, this could be clinically important in light of possible focal therapy for prostate cancer [35, 36]. Overall, this will result in a better quality of care for the patient.

## Conclusion

Digital pathology and 3D reconstructions have the potential to improve dialog between the pathologist and urologist, and, therefore, result in a better treatment selection for urologic patients. For further refinement of analyzing pathology and usage of digital slides, 3D pathology is now on show.

## Electronic supplementary material

Below is the link to the electronic supplementary material.

**Supplementary Figure** **1** a. General pathology workflow. b. workflow with the implementation of digital pathology (TIFF 18958 kb)

## Abbreviations

2D: Two-dimensional
3D: Three-dimensional
H&E: Hematoxylin and eosin
CAD: Computer-aided diagnosis
WSI: Whole slide image
