# Supplementary figures and images for: Histopathology: ditch the slides, because digital and 3D are on show
Source: World J Urol. 2018 Feb 2;36(4):549–55. doi: 10.1007/s00345-018-2202-1 (PMC5871638; doi:10.1007/s00345-018-2202-1)

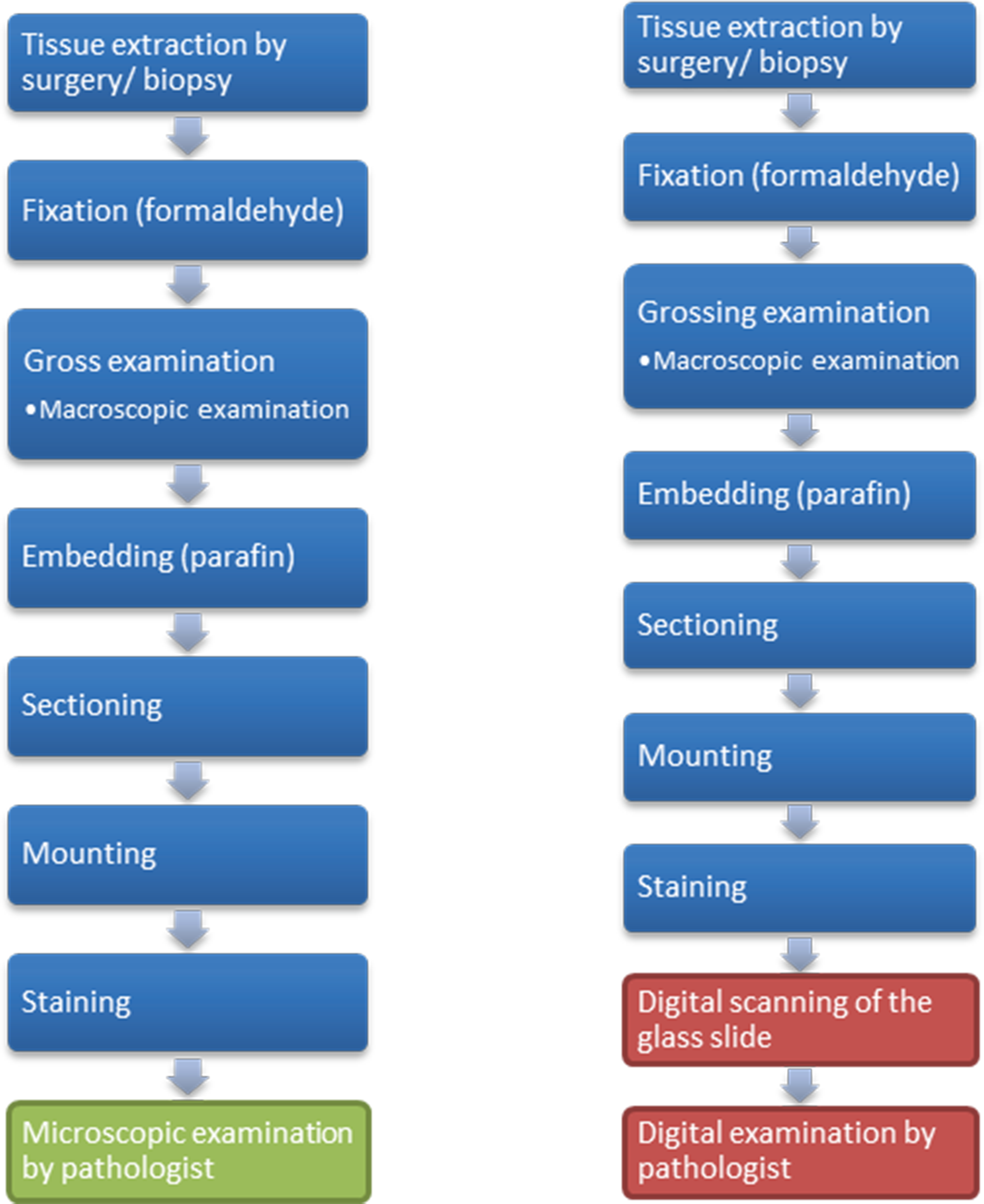

Supplement: Supplementary file 1 — Supplementary Figure 1 a. General pathology workflow. b. workflow with the implementation of digital pathology (TIFF 18958 kb) [file 345_2018_2202_MOESM1_ESM.tif]
